# Processes Contributing to the Maintenance of Flying Phobia: A Narrative Review

**DOI:** 10.3389/fpsyg.2016.00754

**Published:** 2016-06-01

**Authors:** Gavin I. Clark, Adam J. Rock

**Affiliations:** School of Behavioural, Cognitive and Social Sciences, University of New EnglandArmidale, NSW, Australia

**Keywords:** flying phobia, aviophobia, fear of flying, maintenance, anxiety disorders, perceived threat

## Abstract

Flying phobia is a highly prevalent anxiety disorder, which causes sufferers significant distress and life interference. The processes which maintain flying phobia remain poorly understood. A systematic search of the literature was performed to identify what research has been conducted into the processes which may be involved in the fear of flying and whether processes which are believed to maintain other anxiety disorder diagnoses have been investigated in flying phobia. The results of the literature review are presented and related to existing cognitive behavioral theory and research. The results indicate that little research has been conducted into a number of areas considered important in the wider cognitive behavioral literature on anxiety disorders: namely attention, mental imagery, memory, worry, and safety-seeking behaviors. The review proposes a hypothetical model, derived from cognitive behavioral theory, for the processes which may be involved in maintaining flying phobia, and considers a number of areas for future research.

## Introduction

*Flying Phobia* (also referred to as *aviophobia* and *fear of flying*) is an anxiety disorder, which is classified by the Diagnostic and Statistical Manual of Mental Disorders Fifth Edition (DSM-5; American Psychiatric Association, 2013) as a Specific Phobia. Diagnostic criteria for situational Specific Phobia suggest that individuals with flying phobia experience persistent marked fear or anxiety during, or in anticipation of, flying on aeroplanes, which is disproportionate to the actual danger posed by flying. The diagnostic criteria also describes that sufferers actively avoid or endure flying with intense fear or anxiety and that this fear, anxiety, or avoidance causes clinically significant distress or impairment (American Psychiatric Association, 2013).

Flying phobia is considered to have an aversive impact on sufferers' well-being and their functioning related to their profession and social relationships (Van Gerwen and Diekstra, 2000). Indeed, many individuals with flying phobia will avoid placing themselves in flying situations as much as possible (Wilhelm and Roth, 1997). A variety of research has indicated that flying-related anxiety is highly prevalent within the general population, with yearly prevalence estimates ranging from 2.5% to as high as 40% (Ekeberg et al., 1989; Van Gerwen and Diekstra, 2000). The disparity between these upper and lower prevalence estimates may be explained by the fact that lower estimates are associated with research aiming to identify clinically significant phobia whilst higher estimates may be indicative of studies which describe individuals with a self-identified fear of flying (Oakes and Bor, 2010a). The prevalence, in addition to the emotional and functional impact, of flying phobia is highly comparable to research findings relating to other Diagnostic and Statistical Manual of Mental Disorders IV (DSM-IV; American Psychiatric Association, 1994) and DSM- 5 (American Psychiatric Association, 2013) defined anxiety disorders including Social Anxiety Disorder (SAD), Obsessive Compulsive Disorder (OCD), Generalized Anxiety Disorder (GAD), Panic Disorder and Post Traumatic Stress Disorder (PTSD; e.g., Saarni et al., 2007). However, whilst the aforementioned disorders have been subject to a wide array of theoretical discussion and empirical investigation (e.g., Clark, 1999), there has been a relative paucity of research concerning flying phobia.

Cognitive behavioral theory (Beck, 1967, 1976) has provided the basis for the most widely accepted accounts of the etiology and maintenance of anxiety disorders and provides the rationale for the current treatment of choice for anxiety disorders (e.g., NICE, 2011). This theory suggests that individuals with anxiety disorders display a preoccupation with danger and the overestimation of threat of benign stimuli, accompanied by a perception that they will be unable to cope with this potential danger (Beck et al., 1985). The perception of threat is conceptualized as leading to the activation of underlying beliefs and assumptions which give rise to various behavioral and affective responses (e.g., Wells, 1997), which, in turn, may exacerbate and maintain the perception of threat across anxiety disorder diagnoses (e.g., Clark, 1999; Harvey et al., 2004). Reviews of the anxiety disorder literature have highlighted the importance of avoidance and safety-seeking behaviors (Clark, 1999), reasoning and information-processing biases (Harvey et al., 2004), threat-related mental imagery (Hirsch and Holmes, 2007), recollection of anxiety-provoking memories (Hackmann et al., 2000), selective attention toward and/or away from threat (Bar-Haim et al., 2007) and other cognitive vulnerabilities.

Cognitive behavioral therapy (CBT) approaches have been utilized within a highly effective paradigm whereby the detailed clinical investigation of anxiety disorders has led to the development of diagnosis-specific theoretical models of maintenance, with the specified processes of maintenance subjected to empirical tests (Clark, 2004). Such evidence-based theoretical models guide psychological treatments, which aim to modify these putative maintaining mechanisms. This model of theory and treatment development has been applied to disorders including GAD (e.g., Dugas et al., 1998; Robichaud and Dugas, 2006), SAD (e.g., Clark et al., 2006), Panic Disorder (e.g., Clark et al., 1994), and PTSD (e.g., Ehlers et al., 2005). Importantly, however, the mechanisms that maintain flying phobia remain poorly understood (Bogaerde et al., 2012) and have yet to be clearly specified and elucidated within a theoretical model of maintenance.

Oakes and Bor (2010b) highlighted the fact that many established treatment programs for the fear of flying have been developed in the absence of mainstream psychological research and, consequently, risk failing to comprehensively address key processes which maintain distress. Indeed, many flying phobia treatments that are described as “CBT” interventions do not include elements considered fundamental to evidence-based CBT treatments for anxiety disorders (e.g., case formulation, eliciting, and rescripting problematic mental imagery; Clark and Salkovkis, in press).

Whilst a number of psychological interventions have been demonstrated to be helpful in treating flying phobia (e.g., Öst et al., 1997) it is not clear what elements of such treatments impact on flying phobia symptoms. Many patients with flying phobia experience panic attacks during flight situations (Van Gerwen et al., 1997); however, flying phobia treatments do not incorporate elements considered fundamental in the treatment of Panic Disorder (e.g., idiosyncratic formulation, identifying and manipulating safety behaviors; Clark et al., 1994). Consequently, these interventions may not adequately address the difficulties of all individuals with flying phobia. The primary treatment technique for flying phobia is exposure to (real or simulated) flying situations and the disconfirmation of feared catastrophe (Öst et al., 1997), yet many individuals who undertake multiple successful flights continue to experience significant distress (Ekeberg et al., 1989). Indeed, approximately a third of the general population experiences anxiety associated with flying yet only one third of these anxious fliers do not fly at all (Oakes and Bor, 2010a). Collectively, this research suggests that flying phobia is a distressing and prevalent disorder yet the processes which maintain the disorder remain poorly understood. Consequently, the current conceptualization of flying phobia and existing psychological treatments may fail to adequately address the processes contributing to the maintenance of anxiety responses associated with flying phobia.

### Aim of review

The processes that maintain flying phobia have received relatively little attention compared to the wealth of empirical studies that have examined the processes involved in maintaining other anxiety disorder diagnoses. It would, therefore, be of value to evaluate flying phobia in the context of the wider anxiety disorder literature. A number of common processes of maintenance have been demonstrated across anxiety disorders (McManus et al., 2010), and anxiety disorders have been argued to share a common core pathology (Barlow et al., 2004). Reflecting such commonalties, research paradigms employed in the investigation of specific anxiety disorders (e.g., regarding selective attention processes) have been used to inform the understanding and investigation of other anxiety disorder diagnoses (e.g., Wolitzky and Telch, 2009). This review will, therefore, aim to describe the processes that may maintain flying phobia by considering the following questions:

1. What investigation has taken place concerning flying phobia in regards to the processes of maintenance that have been identified across anxiety disorder research?
2. Where no research has taken place, what hypotheses can be formulated regarding the processes which maintain flying phobia based on research findings concerning other anxiety disorders?
3. What areas for future research are suggested by the current status of the flying phobia literature?

Given that much of the research concerning anxiety disorders has taken place in the context of the DSM-IV (American Psychiatric Association, 1994) categorization, this review will consider OCD and PTSD-related research even though these disorders exist in diagnostic categories outwith anxiety disorders within the DSM-5. Our rationale for this decision is based on the significant degree of overlap between constructs and processes of maintenance that has been demonstrated between these disorders and other anxiety disorder diagnoses (Harvey et al., 2004; McManus et al., 2010), which may be considered pertinent in the understanding of flying phobia.

### Literature search procedure

Following the procedure outlined by Amiot and Bastian (2014) our literature search involved generating a pool of potentially pertinent articles and, subsequently, identifying a subset of articles for inclusion in this review. A series of literature searches were conducted of title and abstract fields, published between 1960 and August 2015, in online databases (i.e., Google Scholar, Medline, Embase, and PsychInfo) by pairing the terms *flying, flying phobia, flight phobia, aviation phobia, aviophobia, fear* and *fear of flying* with search terms related to the maintenance processes which have been identified as common maintenance mechanisms in reviews of the anxiety disorder literature (e.g., Clark, 1999; Harvey et al., 2004; McManus et al., 2010). The following search terms were used: *attention, attention bias, attentional bias, images, imagery, mental imagery, bias, cognitive bias, cognitive biases, information processing bias, reasoning, misinterpretation, interpretation, belief*, *metacognition, behaviour, safety behaviour, safety-seeking behaviour, escape, avoidance, counter-productive strategies, emotion regulation, emotion regulation strategies, anxiety sensitivity, triggers, phobic stimuli, threat, cue-reactivity, memory, memories, memory bias, recall, intolerance of uncertainty*, and *worry*. This search strategy yielded 529 articles, book chapters and books. Subsequently, title and abstracts were inspected in order to determine whether they described empirical studies related to: (i) flying phobia; and (ii) variables that may be involved in the maintenance of flying phobia.

Studies were deemed pertinent if they included the main topic area (i.e., flying phobia) and one or more outcomes of interest (e.g., memory and/or attention). Articles were listed as non-pertinent if they only reported on one topic of interest (e.g., *flying phobia* or *attention*). Studies were also excluded if they examined the prevalence of the disorder or the efficacy of psychological interventions for flying phobia solely in terms of changes in subjective anxiety. In total, 24 manuscripts were deemed pertinent and were included within this review.

The empirical articles identified are described in Table 1 and are organized under each of the broad transdiagnostic areas of maintenance identified across anxiety disorders for which at least one study had been carried out in relation to flying phobia. Before commencing the review it is important to note that whilst the review of the flying phobia literature was conducted systematically, it was beyond the scope of this review to examine research from the wider anxiety disorder field in a systematic manner. Consequently, research conducted across other anxiety disorders was identified within non-systematic searches and through utilizing meta-analyses and reviews (e.g., Bar-Haim et al., 2007), and is cited in order to illustrate the role of maintenance processes across anxiety disorders rather than presenting an exhaustive overview of the wider anxiety disorder literature.

**Table 1:** **Summary of empirical research on maintenance processes in flying phobia**.

**Study**	**Country**	**Population**	**Sample size and characteristics**	**Design**	**Focus of study**	**Findings**
**SELECTIVE ATTENTION**						
van Almen and van Gerwen, 2013	Netherlands	General population, individuals who completed an open-access questionnaire on the website of a foundation which offers treatment for flying phobia	*N* = 9116 Mean age 34.37 years (*SD* = 11.80)	Correlational	Evaluating the relationship between fear of flying (as measured by FAM) and voluntary allocation of attention toward (monitoring) or away (blunting) from threatening information as measured by *Miller Behavioral Style Scale* (consists of descriptions of four hypothetical uncontrollable stressful situations).	Individuals who reported higher levels of monitoring of threat in response to hypothetical stressful scenarios reported higher levels of flying anxiety (as measured by FAM). (Note: the self-reported voluntary allocation of attention may arguably be classified as a cognitive strategy rather than reflecting selective attentional processes).
**PERCEIVED THREAT/FEARED OUTCOME**						
Hawkins-Gilligan et al., 2011	USA	University students, recruited from university class-rooms	*N* = 109	Correlational	Evaluating whether flying-embedded fears (i.e., fears regarding other stimuli associated with flying such as agoraphobic or height-focussed fears), as measured by the *Fear Survey Schedule*, and individuals' flying-specific conditioning experiences (including direct, observational and verbal) predicted fear of flying symptomology as measured by FAS subscales and an adapted version of the *Fear Survey Schedule*.	All 5 fear of flying measures were predicted by at least one flying-embedded fear (relating to fears surrounding social outcomes, heights, agoraphobic fears and fears relating to water). Previous conditioning experiences only predicted one of the 5 fear of flying measures and this was labeled as “weakly” predicting this variable. The authors concluded that the results suggest that the fear of flying is based on several flight-embedded innate fears rather than fears conditioned through learning.
McNally and Louro, 1992	USA	Flying phobics, based on DSM-III-R criteria for specific phobia and individuals with panic disorder with agoraphobia (PDA; based on DSM-III-R criteria) who sought help for fear of flying	*N* = 34, 17 flying phobics, Mean age 36.12 (*SD =* 6.73) 17 PDA participants Mean age 26.82 years (*SD =* 8.58)	Structured interviews and descriptive statistics	Evaluating reasons for flight avoidance and feared outcomes in a group of individuals with simple phobia and those with PDA who sought help for fear of flying.	Individuals with specific phobia of flying reported fears around external threat (plane crashing) as primary concerns. PDA participants reported that their fears primarily related to internal threat (panic attacks and consequences).
Möller et al., 1998	South Africa	Flying phobics, based on scoring 5 or more out of 10 on the *Questionnaire on Attitudes Toward Flying*	*N* = 22, 15 flying phobics, Mean age 47.7 years, 7 non-fearful flyers, Mean age 44.8 years	Experimental	Evaluating whether the fear of flying is associated with the reporting of irrational evaluative beliefs when faced with hypothetical flying scenarios. Fearful and non-fearful fliers were presented with six threatening and six non-threatening flying scenarios as part of Articulated Thoughts in Simulated Situations procedure.	The flying phobic group reported more irrational thoughts and higher subjective anxiety ratings across experimental scenes compared to the non-fearful fliers. Irrational thoughts reflected an overestimation of the seriousness of threat (“awfulizing”) and underestimating perceived ability to cope (“low frustration tolerance”).
Van Gerwen et al., 1997	Netherlands	Flying phobics, based on self-referral to fear of flying program	*N* = 419, Mean age 40.9 years, (*SD* = 10.4)	Semi-structured interview and self-report questionnaires	Evaluating clinical characteristics of flying phobia including presence of panic attacks, situational avoidance, flight history, level of flight anxiety and primary concerns when flying.	Identified four subtypes of flying phobia: (1) Individuals with no panic attack symptoms primarily concerned with aircraft accidents and the need for situational control; (2) individuals who fear loss of control over themselves or social anxiety and pay significant attention to somatic sensations; (3) individuals whose fears center on water and/or claustrophobia and agoraphobia and report panic attacks associated with flying; and (4) individuals who primarily present with fears reflecting acrophobia (irrational fear of heights).
Wilhelm and Roth, 1997	USA	Flying phobics, based on DSM-III-R criteria, individuals with Panic Disorder and Agoraphobia (PDA), individuals with a history of Panic Disorder with Agoraphobia (PDH) and non-phobic control group, individuals who responded 0 or 1 out of 10 when rating their flying anxiety, recruited from the general population	*N* = 87, 37 flying phobics, Mean age 44.0 years (*SD* = 10.5), 18 PDA participants, Mean age 50.3 years (*SD* = 10.0) 11 PDH participants Mean age 44.5 years (*SD* = 11.6) 21 non-phobic controls, Mean age 44.1 years (*SD* = 13.7)	Correlational and use of structured interview	Evaluating clinical characteristics of flying phobia including onset of symptoms, flight history and experience of traumatic flying events and feared outcomes/concerns when flying.	Flying phobics, PDA and PDH participants reported equivalent concerns about external dangers (e.g., plane crash, pilot making mistake), though PDA and PDH participants reported greater concern regarding internal or social anxiety stimuli during flights.
**COGNITIVE BIAS**						
Mühlberger et al., 2006	Germany	Flying phobics and Spider phobics (based on DSM-IV criteria)	*N* = 34, 17 spider phobics, Mean age 27.4 years, (*SD* = 9.3) 17 flying phobics, Mean age 44.2 years (*SD* = 9.6 years)	Experimental	Examined presence of expectancy and covariation bias by exposing participants to pictures of spiders, flight accidents and neutral cues followed by either a startling noise or no stimulus whilst measuring skin conductance responses, startle response and event-related brain potentials.	Flying phobics and spider phobics displayed a threat-disorder-specific expectancy bias and skin conductance responses but only spider phobics demonstrated a disorder-specific covariation bias.
Pauli et al., 1998	Germany	Flying phobics, based on high FFS scores and non-phobic control group based on low FFS scores	*N* = 28, 14 flying phobics, Mean age 32.8 years (*SD* = 7.7) 14 non-flying-phobics, Mean age 30.7 years (*SD* = 6.9)	Experimental	Examined covariation bias in flying phobia through pairing fear-relevant stimuli (slides of aeroplane crash sites) or fear-irrelevant stimuli (slides of aeroplanes in flight or mushrooms) and electric shocks.	Flying phobics demonstrated a covariation bias and reported higher covariation estimates for fear-relevant slides and shocks than fear-irrelevant slides and shocks, relative to non-flying phobics.
Vriends et al., 2012	Switzerland	Flying phobics, based on DSM-IV criteria, and non-phobic control group recruited from university	*N* = 72, 33 flying phobics, Mean age 36.4 years (*SD* = 9.3) 39 “healthy controls”, Mean age 36.1 years (*SD* = 11.1)	Experimental	Evaluating associative learning in flying phobia. Participants viewed a series of distracters interspersed with pairings of novel objects with frightening and pleasant stimuli.	Flying phobics demonstrated a stronger conditioning effect and subjective ratings of conditioned stimuli as frightening compared to healthy controls.
**RESPONSE TO PHOBIC CUES/TRIGGERS**						
Bogaerde and De Raedt, 2013	Belgium	Flying phobics, based on Mini International Neuropsychiatric Interview and non-phobic control group recruited from non-phobic passengers on exposure flight	*N* = 103, 54 flying phobics, Mean age 40.22 years 49 non-phobic controls, Mean age 41.85 years	Experimental	Evaluating oxygen saturation (SpO_2_), anxiety and subjective somatic associations of flying phobics and controls on a plane at ground level and at cruising altitude.	Both groups demonstrated a decrease in SpO_2_ from ground level to cruising altitude. Flying phobics reported more somatic sensations and elevated anxiety at ground level. At cruising altitude flying phobics reported significantly greater somatic sensations but anxiety was no longer significantly greater than controls.
Bornas et al., 2004	Spain	Flying phobics, based on scoring more than 1.5 standard deviations above the mean on the FFQ in a sample of 230 undergraduate students. Non-phobic control group based on scoring the mean plus or minus one standard deviation on the FFQ.	*N* = 30, 15 flying phobics, 15 non-phobic controls, Mean age of total initial sample from which participants were selected (*N* = 230) 22 years (*SD =* 3.2)	Experimental	The study evaluated self-implication (self-rated engagement with stimulus) during simulated exposure to flight-related pictures, sounds or pictures and sounds combined. Measures of heart-rate variability (reflecting degree of autonomic flexibility and ability to respond to environmental demands), subjective anxiety and subjective rating of self-implication were recorded.	The study found the phobic group demonstrated significantly higher measures of anxiety than the non-phobic group. Flying-related sounds elicited greater anxiety than flying-related pictures. There were no significant group differences in subjective ratings of self-implication (whether reflecting attentional deployment or cognitive engagement with stimulus) and no correlation between self-implication and anxiety. Flying phobics who displayed low heart-rate variability displayed higher self-implication in relation to flight-related sounds than flying phobics with high heart-rate variability.
Bornas et al., 2006	Spain	Flying phobics, based on DSM-IV criteria	*N* = 61, Mean age 39.07 (*SD* = 11.24)	Experimental	Measured individual heart-rate response at baseline, during a paced breathing task and then on exposure to a video of a flight scenario.	Participant heart-rate increased from baseline to paced breathing exercise and decreased from paced breathing to exposure to flight video.
Busscher et al., 2010	Netherlands	Flying phobics, based on DSM-IV criteria and non-phobic control group recruited from the general population	*N* = 163, 127 flying phobics, Mean age 40.4 years (*SD* = 11.0) 26 non-phobics, Mean age 43.4 (*SD* = 13.5)	Experimental	Evaluating participant responses to a neutral video, phobic stimulus (video depicting a flight-scenario) and a recovery period across phobic and non-phobic groups. Measures of heart-rate, subjective anxiety, and cardiac autonomic response were taken.	Significantly higher subjective anxiety was reported by the phobic group on exposure to the flight-related stimulus but group differences were not found on physiological measures. Within the phobic group heart-rate and cardiac response were moderately strongly coupled with subjective anxiety.
**BEHAVIORS/COPING RESPONSE**						
Girodo and Roehl, 1978	Canada	Flying phobics, identified from an undergraduate sample based on likert scale indicating flight apprehension	*N* = 56, Mean age 21 years	Experimental	Evaluating the impact of training participants in two cognitive coping strategies (coping self-talk and information regarding what would occur when flying) on self-reported anxiety before, during and following a flight in which an unexpected negative event occurred. Participants were split into four groups: coping self-talk, information, combined and control.	No differences were recorded between subjective anxiety between groups when flying, individuals who had been trained in coping self-talk reported less anxiety associated with an unexpected negative flying event.
Kraaij et al., 2003	Netherlands	Flying phobics, based on DSM-IV criteria, who sought treatment for fear of flying	*N* = 261, Mean age = 38.4 years (*SD* = 10.14)	Correlational	Evaluating the cognitive coping strategies that individuals with fear of flying report using to regulate their emotions during a flight experience based on responses to the CERQ. FAS and FAM were also administered.	Participants reported using strategies including focussing on planning, rumination, and putting in perspective. Greater use of self-blame, rumination, acceptance and/or catastrophizing were all associated with higher levels of subjective anxiety associated with flying.
Nousi et al., 2008	Netherlands	Flying phobics who sought treatment for fear of flying, grouped according to: (1) those who had never flown; (2) those who had flown and experienced no adverse incidents; and (3) those who had experienced adverse or traumatic flights.	*N* = 489 (from an initial sample of 2001) 174 in Group 1, Mean age 46.63 years (*SD* = 13.4) 200 in Group 2, Mean age 39 =.63 years (*SD* = 10.8) 115 in Group 3, Mean age = 28.32 (*SD* = 10.1)	Experimental	Evaluating the prevalence and characteristics of individuals (including flying anxiety as measured by FAS and FAM) with different flying histories and their predictive value of flying histories in treatment outcome. The evaluation of a group of fearful fliers who had never flown may be used to infer the impact of situational avoidance.	Participants who had never flown reported higher levels of fear of flying and general anxiety and demonstrated greater reduction in anxiety following treatment. The authors concluded that participants who had never flown before may have fear which reflect more generalized avoidance tendencies and may over-predict the magnitude and intensity of their anxiety.
Wilhelm and Roth, 1997	USA	Flying phobics, based on DSM-III-R criteria, individuals with Panic Disorder and Agoraphobia (PDA), individuals with a history of Panic Disorder with Agoraphobia (PDH) and non-phobic control group, individuals who responded 0 or 1 out of 10 when rating their flying anxiety, recruited from the general population	*N* = 87, 37 flying phobics, Mean age 44.0 years (*SD* = 10.5), 18 PDA participants, Mean age 50.3 years (*SD* = 10.0) 11 PDH participants Mean age 44.5 years (*SD* = 11.6) 21 non-phobic controls, Mean age 44.1 years (*SD* = 13.7)	Correlational and use of structured interview	Evaluating clinical characteristics of flying phobia including onset of symptoms, flight history and experience of traumatic flying events and feared outcomes/concerns when flying.	Individuals with flying phobia reported utilizing avoidance, alcohol and medication to attenuate anxiety symptoms associated with flying.
**ANXIETY SENSITIVITY**						
Busscher et al., 2010	Netherlands	Flying phobics, based on DSM-IV criteria, who sought treatment for fear of flying and non-phobic control group recruited from the general population	*N* = 163, 127 flying phobics, Mean age 40.4 years (*SD* = 11.0) 26 non-phobics, Mean age 43.4 (*SD* = 13.5)	Experimental	Evaluating participant responses to a neutral video, phobic stimulus (video depicting a flight-scenario) and a recovery period across phobic and non-phobic groups. Measures of heart-rate, subjective anxiety, and cardiac autonomic response were taken.	Anxiety sensitivity did not moderate the relationship between subjective anxiety and physiological measures of anxiety in the flying phobic group (additional findings noted above).
Busscher et al., 2013	Netherlands	Flying phobics, based on DSM-IV criteria, who sought treatment for fear of flying	*N* = 50, Mean age 28.4 years (*SD* = 10.6)	Experimental	Evaluating the relationship between reported flight anxiety, physiological arousal (as measured by heart-rate, respiratory sinus arrhythmia and pre-ejection period) and anxiety sensitivity, during an exposure to a real flight scenario on an aeroplane.	Results indicated that anxiety sensitivity moderated the relationship between changes in physiological arousal and self-reported flight anxiety but not between self-reported somatic sensations and flight anxiety.
Busscher et al., 2015	Netherlands	Flying phobics who sought treatment for fear of flying	*N* = 79, Mean age 40.4 (*SD* = 11.0)	Experimental	Evaluating emotional processing theory in relation to flight phobia by investigating whether success of exposure therapy (i.e., future reduction in anxiety) is predicted by activation of subjective and physiological fear responses and their within-session and between-session habituation. Participants underwent measurements of self-reported and physiological anxiety (including heart-rate, respiratory sinus arrhythmia) during within-session habituation and between session-adaption to exposure to flying-related stimuli, simulated flight and a real flight.	Within-session habituation and between-session adaption was recorded on physiological and subjective measures during exposure but this did not predict treatment outcome. The authors concluded that results provided only weak support for emotional processing theory.
Bogaerde and De Raedt, 2008	Belgium	General population, recruited from university undergraduate population	*N* = 160, Mean age 31 years	Correlational	Evaluating the role of anxiety sensitivity in the fear of flying by administering the FAS, FAM and *Anxiety Sensitivity Index*.	A stronger relationship between in-flight anxiety and somatic sensations was found for individuals with high anxiety sensitivity relative to those with low anxiety sensitivity, suggesting that anxiety sensitivity moderates this relationship.
Bogaerde and De Raedt, 2011	Belgium	Flying phobics, based on DSM-IV criteria, and non-phobic control group recruited from non-phobic passengers on exposure flight	*N* = 103, 54 flying phobics, Mean age 40.2 years 49 non-phobic controls, Mean age 41.9	Correlational	Evaluating the role of anxiety sensitivity by measuring anxiety sensitivity, subjective anxiety on a visual analog scale, flying anxiety (on FAS) and subjective bodily sensations in participants immediately before taking a flight.	Anxiety sensitivity was found to moderate the relationship between somatic sensations and flying phobia symptoms, with somatic sensation predicting flight anxiety in high anxiety sensitivity individuals but not in low anxiety sensitivity individuals.
**MEMORY**						
Bogaerde et al., 2012		Flying phobics, based on scoring 4 on a 1-to-4 likert scale of flying anxiety, and non-phobic control group both recruited from undergraduates	*N* = 25, 12 flying-phobics, mean age 18.7 years (*SD* = 0.9). 13 non-phobic controls, mean age 18.8 years (*SD* = 2.8)	Experimental	Investigating threat in fear of flying through measuring participant free recall of external vs. internal threat words and neutral words in a dichotic listening task.	Flying phobics displayed greater recall of internal threat words (i.e., consequences of anxiety sensations) than non-flying phobics. No group differences were found for external threat words or neutral stimuli.
**WORRY**						
Aitken et al., 1981	UK	Flying phobic air-crew (RAF aircrew referred for treatment) and non-flying phobic air-crew controls	*N* = 40 20 flying phobics, 28.6 years (*SD* = 5.5) 20 Non-flying-phobics 30.4 years (*SD* = 5.1)	Experimental and Correlational	Aimed to compare the characteristics of flight phobic air-crew compared to non-flight phobic air-crew by administering self-report worry questionnaire, clinical interview, psychophysiology measures (heart-rate and galvanic skin response to repeated auditory tone) and a battery of personality inventories.	Results indicated no significant difference on measures of personality but the flying phobic group displayed greater fluctuations in skin conductance and habituated less to a repeated auditory tone. The flying phobic group reported greater worries concerning flying and also concerning their marital partners. More of the flying phobic group also reported a family history of a flying-related trauma.
Bergstrom and McCaul, 2004	USA	General population, recruited from university psychology undergraduates	*N* = 115, Mean age 21.3 years	Correlational	Assessing worry as a predictor of flying-related decision making 34 days after the September 11th terrorist attacks in the USA.	Subjective worry predicted estimates of one's own and others' willingness to fly.
Martinussen et al., 2011	Norway	General population, recruited from passengers in arrivals hall at airport	*N* = 270, Mean age 35.1 years (*SD* = 14.5)	Correlational	Assessing fear of flying (using FAS), positive emotions toward air travel and stress/worry related to check-in and security checks.	Flying-related anxiety was predicted by gender (female), recent flying experience, and stress/worry regarding check-in and security checks.

Wilhelm and Roth (1997) and Busscher et al. (2010) are described under two headings as they report outcomes in two areas of interest but are single publications. Mean age and standard deviation of sample are reported to two decimal places where available. Our search did not identify any investigation of mental imagery, intolerance of uncertainty or any other processes implicated in the maintenance of anxiety disorders. The organization under each area of maintenance does not connote mutual exclusivity in study focus/outcome, e.g., selective attention may be hypothesized to be demonstrated in the higher levels of recall of internal threat words in the study by Bogaerde et al. (2012). FAS, Flight Anxiety Situations Questionnaire (Van Gerwen et al., 1999); FAM, Flight Anxiety Modality Questionnaire (Van Gerwen et al., 1999); FFQ, Fear of Flying Questionnaire (Bornas et al., 1999); FFS, Fear of Flying Scale (Johnsen and Hugdahl, 1990); CERQ, Cognitive Emotion Regulation Questionnaire (Garnefski et al., 2002).

## Perceived threat and related cues

The appraisal of a particular stimulus or situation as threatening is considered to be a fundamental process in the experience and exacerbation of anxiety (Clark, 1999). Stimuli which cue threat-perception, also referred to as *triggers*, vary according to feared outcome, threat-object and the extent to which this threat has generalized to more diffuse stimuli (Dunsmoor et al., 2009) e.g., an initial fear-response to turbulence ultimately being cued by simply seeing a plane. Individuals with flying phobia report high levels of anxiety associated with all stages of flying including anticipation, boarding, in-flight and landing, with the highest self-reported anxiety ratings being associated with take-off and bad weather/turbulence (Wilhelm and Roth, 1997). Wilhelm and Roth conducted an evaluation of the clinical characteristics of flying phobia and reported that flight-related concerns in flying phobia can be typically categorized into three factors: external danger, internal/social danger, and flight hassles. Concern reflecting external danger may include fears around accidents, the aeroplane crashing, mechanical complications, and threatening weather conditions. Concerns associated with internal/social danger may include concerns around the consequences of other people being critical and humiliating, appearing mentally ill, losing emotional control of oneself, bodily discomfort, and anxiety leading to psychological or physiological catastrophe. The final category reflects worries regarding organizational issues (e.g., delays or missing luggage) and has not featured in the majority of descriptions of the primary fears of flying phobics. An additional concern relates to the likelihood of terrorist activity when flying, and many individuals report increases in worry and avoidance of flying following reports of plane-related terrorist incidents (Bergstrom and McCaul, 2004; Gigerenzer, 2004).

Due to the variety of feared outcomes identified in flying phobia, flying phobics have been described as a heterogeneous group, and it has been suggested that the distress experienced by flying phobics when flying may be the manifestation of one or more other anxiety disorders (e.g., McNally and Louro, 1992; Van Gerwen et al., 1997; Wilhelm and Roth, 1997). Whilst the research carried out over the past three decades into the fear of flying has consistently conceptualized flying phobia as a form of specific phobia (as described by the DSM-IV; American Psychiatric Association, 1994) it has been suggested that, in addition to more general fears around physiological and situational dyscontrol, the various fears reported by flying phobics are consistent with the concerns reported in disorders such as claustrophobia, agoraphobia, acrophobia and SAD (McNally and Louro, 1992; Van Gerwen et al., 1997; Wilhelm and Roth, 1997; Busscher et al., 2010). A study by Hawkins-Gilligan et al. (2011) suggested that fear of flying symptoms in a sample of undergraduates were better predicted by flying-embedded fears (i.e., fears regarding other stimuli associated with the flying experiences such as heights or agoraphobic fears) than fears conditioned by negative flying experiences. There is evidence that up to 59% of individuals with a specific phobia of flying will meet criteria for another anxiety disorder within their lifetime (Depla et al., 2008), perhaps suggesting that processes which contribute to the anxiety experienced by those with a fear of flying may manifest across a number of contexts. Regarding the potential impact of comorbidity, McNally and Louro (1992) suggested that distinct presentations of flying phobia exist where the comorbid presentation of agoraphobia will lead to concerns around the occurrence and consequences of panic sensations and those without agoraphobia will report greater concerns regarding external flying-related events (e.g., crashing).

Regardless of these diagnostic considerations a number of stimuli have been demonstrated to cue threat-perception and anxiety response in flying phobia including a variety of external stimuli associated with flying (e.g., planes, safety briefings, cabin announcements) and interoceptive information (e.g., heart-rate, breathing difficulties; van Almen and van Gerwen, 2013). However, the nature of the internal and external cues that most typically trigger threat perception in flying phobia has yet to be fully understood.

A number of studies have investigated flying phobics' response to flying-related stimuli including plane-crash-related images, flight-related sounds, videos of flights, descriptions of aversive flight scenarios, virtual flight simulation and *in-vivo* exposure to actual flights (e.g., Möller et al., 1998; Bornas et al., 2006; Mühlberger et al., 2006; Busscher et al., 2013). This research has found that flying phobics report increases in subjective distress and has demonstrated increases in physiological distress (e.g., skin conductance, increased heart rate and increased cardiac autonomic activity) in response to such stimuli (e.g., Wilhelm and Roth, 1997; Busscher et al., 2010). Interestingly, such research has found greater anxiety reported in response to flying-related sounds than images in simulated laboratory environments (e.g., Bornas et al., 2004). It is notable that little research has investigated individuals' cognitive responses to such cues and which variables might influence this subjective distress, other than the cognitive vulnerability of anxiety sensitivity (described below).

A number of reasons may account for a diverse range of cues that can elicit flight-related anxiety. Traditional classical conditioning models suggest that the pairing of a neutral stimulus (CS; e.g., flying or specific flying-related stimuli), with an aversive event (UCS), which naturally elicits fear (UCR), will lead to that stimulus eliciting future fear and associated behavior (e.g., situational avoidance; Bogaerde et al., 2012). However, many people who have a fear of flying have never experienced threatening or traumatic flying events and do not report more flying-related traumatic events than non-flying phobics (Wilhelm and Roth, 1997). One explanation offered by Bogaerde et al. (2012) is that interoceptive conditioning may occur whereby an interoceptive stimulus (e.g., the sensation of breathlessness) becomes associated with a conditioned fear response. It has been noted that there are a multitude of sources of interoceptive sensations that may be elicited by the flying experience; breathlessness, light-headedness and increased heart rate are all associated with lower oxygen saturation levels at high altitude (Bogaerde and De Raedt, 2013), and mechanical vibrations, motion and acceleration all impact upon the vestibular system (Jaffee, 2005). It has also been suggested that individuals with anxiety disorders may be more prone to experience somatic distress associated with high altitude (Roth et al., 2002). Consequently, Bogaerde and De Raedt (2013) suggested an alternative conditioning model where internal sensations may come to be the primary source of fear when somatic symptoms (UCS; triggered by the environmental effects of the flying experience such as hypoxia) may be experienced as more pronounced by some individuals, and thus, result in a fear response (UCR). The association of flying (CS) with aversive interoceptive sensations would, therefore, lead to flying anxiety (CR).

Summarising the onset of flying phobia in their interview study, Wilhelm and Roth (1997) report that, for many individuals, the fear of flying “apparently began with a rise in anxiety of flying that was either triggered internally or was a transitory overreaction to a minor external event” (p. 258). It is notable that experimental evidence suggests that individuals with flying phobia demonstrate a stronger conditioning effect when neutral stimuli are paired with aversive stimuli (not associated with flying) compared to non-flying phobics (Vriends et al., 2012). This finding might suggest that individual differences in preparedness for associative learning (i.e., in the pairing of external or internal neutral conditioned stimuli with aversive stimuli) may contribute to the development of flying phobia.

Armfield (2006) identified a number of variables crucial to explaining the characteristics of fear objects in anxiety disorders, which include danger, unpredictability, uncontrollability, and perceived vulnerability. These variables may be seen to be pertinent in the reported fears of flying phobics. Fears concerning flying-related catastrophe such as crashes, severe turbulence or acts of terrorism have been reported by flying phobics (e.g., Wilhelm and Roth, 1997; Bergstrom and McCaul, 2004). It might be hypothesized that the occurrence of turbulence, aircraft motion and associated anxiety may be perceived as unpredictable and uncontrollable. Uncontrollability may also be relevant to fears regarding physiological and cognitive dyscontrol, which are common amongst flying phobics (Busscher et al., 2013). The process by which a given stimulus cues threat perception in flying phobia remains to be understood.

## Attention

*Selective attention* may be defined as “a process by which specific stimuli, within the external and internal environment, are selected for further processing” (Harvey et al., 2004, p. 26). Attentional bias toward threat-related cues is believed to play an important role in the maintenance of anxiety disorders through increasing awareness of triggers, which leads to increases in perceived threat (Beck et al., 1985). The preferential allocation of attention in reference to threat-related cues has been evaluated in a large number of research studies across anxiety disorder diagnoses (Bar-Haim et al., 2007).

It has been suggested that attentional bias has the potential to play a role in the etiology and maintenance of flying phobia (Oakes and Bor, 2010a) and may be associated with cognitive biases, which contribute toward promoting the perception of threat in flying phobia (Wilhelm and Roth, 1997). However, to date, there has been very limited consideration of the role of selective attention in maintaining flying phobia and we identified only one study that directly investigated attention in relation to the fear of flying. This single study, conducted by van Almen and van Gerwen (2013), reported that increased monitoring of threat cues (quantified by a general self-report measure of responses to hypothetical non-flying stressful situations) was associated with higher self-reported flight anxiety. This study was not specific to flying-related material and measured participants' perception that they would engage in the intentional deployment of attention (i.e., monitoring or avoiding threat-relevant information), which may be conceptualized as being distinct from the automatic allocation of attention toward threat stimuli observed across anxiety disorders. Consequently, the role of attentional processes in relation to fear-relevant flying situations cannot be discerned from this research. No evaluation of attentional bias toward flight-related threat stimuli was identified. A study by Bornas et al. (2004) reported that the degree to which individuals rate their “self-implication” (i.e., engagement) with an experimental fear-relevant stimulus was not found to be correlated with anxiety response. However, the sample size of 15 flying phobic and 15 non-flying phobic undergraduate students and lack of operationalization of what self-implication connotes (e.g., whether reflecting attentional deployment or cognitive engagement with the stimulus), limits the extent to which any inferences can be drawn from this study regarding attentional deployment. An attention bias toward threat may be tentatively inferred from the finding that individuals with flying phobia demonstrate greater recall for threat-related words (i.e., concerning aversive physiological outcomes) on a dichotomous listening task compared to a control sample (Bogaerde et al., 2012). This result may suggest that a selective attentional bias toward interoceptive information associated with anxiety sensations (i.e., conceptualized as attention toward *internal threat*; Olatunji et al., 2007) may exist. This is consistent with reports from clinical interviews that suggest that certain individuals with flying phobia closely monitor their somatic symptoms (Van Gerwen et al., 1997).

van Almen and van Gerwen (2013) noted that due to the heterogeneous phobic characteristics of flying phobia across individuals the characteristics of selective attention and attentional bias may be difficult to pinpoint. A wide body of research has demonstrated that attention bias toward threat is reliably demonstrated within anxiety disorders by its manifestation across a number of experimental paradigms and experimental conditions (for a review see Bar-Haim et al., 2007). However, the perceptual paradigms commonly utilized in the study of other anxiety disorders have yet to be applied in reference to flying phobia (e.g., emotional stroop, dot-probe paradigm and the emotional spatial cuing paradigm with the use of threat-related words and naturalistic stimuli). Research across anxiety disorders has demonstrated that the majority of individuals with an anxiety disorder display attentional bias toward *external threat-relevant stimuli* as well as toward anxiety sensations (i.e., *internal threat;* Kampman et al., 2002). The robustness of this attentional bias prompted Bar-Haim et al. to conclude that, “diminishing returns [are] to be expected from further studies that only focus on establishing the presence of a threat-related bias in anxious groups” (p. 15). It would, therefore, be reasonable to hypothesize that attentional bias toward threat in flying phobia exists. However, the identity of external threat-relevant stimuli subject to this bias across flying phobics has not been investigated and the relative impact of internal and external attentional bias regarding anxiety remains unknown.

In addition to attentional bias toward threat in anxiety disorders there is some evidence of selective attention *toward safety* (e.g., escape; Thorpe and Salkovskis, 1998), and, similarly, *away from external threat* (e.g., Chen et al., 2002). These biases are hypothesized to reflect a strategy more amenable to consciousness at a later stage of attention processing (i.e., where attention toward threat is considered automatic and not a conscious strategy) and may be determined by functional consequence (i.e., whether threat can be avoided or neutralized; Clark, 1999). It is notable that in the case of flying phobia the feared situation precludes escape once in-flight and it is not clear how, and to what extent, individuals can direct attention away from internal and external threat cues.

Harvey et al. (2004) suggested numerous ways whereby attention may maintain a psychological disorder, which may be relevant to flying phobia. These include: (i) selective attention toward a concern-related stimulus (e.g., anxiety sensations, interoceptive information about turbulence) may miss information that may lead to the disconfirmation of maladaptive beliefs (e.g., safety cues); (ii) increased attention to internal stimuli may increase internal attributions for events (e.g., confirming the reality of threat based on anxiety sensations); (iii) attention bias may lead to selective encoding of information consistent with negative perceptions and beliefs (e.g., toward disruption of the vestibular system where signals of motion may confirm that the plane is unstable and in danger of losing control); and (iv) automaticity of attention may lead to individuals interpreting this as evidence of cognitive dyscontrol, particularly where they have attempted to avoid or suppress such stimuli.

Given the suggestion that flying phobia may be associated with fears regarding emotional and cognitive dyscontrol (e.g., Busscher et al., 2013), the evaluation of attentional bias toward internal vs. external threat, coupled with attention deployed as an intentional strategy, would seem of significant importance in informing treatment and potentially differentiating subgroups of sufferers. An example of why this could be pertinent can be seen in the treatment of SAD (e.g., Clark et al., 2006), which focuses predominantly on working with attentional bias toward internal threat and re-evaluating interoceptively-derived perceptions in order to attenuate perceived threat and distress (e.g., Pineles and Mineka, 2005). This remains an area which is poorly understood in flying phobia and which is not explicitly addressed within existing treatments. Clearly, more work is needed in order to evaluate individual attentional responses to specific triggers in flying phobia and how these responses contribute to the appraisal of threat.

## Cognitive errors and reasoning biases

As noted above, flying phobics report a number of fears regarding negative outcomes related to flying including the physiological or psychological consequences of escalating anxiety symptoms, the lack of situational control, and plane-related catastrophe such as crashing (Van Gerwen et al., 1997; Wilhelm and Roth, 1997). Given the extremely low probability of air disasters (Oakes and Bor, 2010a) or anxiety-induced negative outcomes (e.g., Salkovskis et al., 1996), such fears, when experienced as an immediate threat, can be considered to reflect the overestimation of threat and, where they pertain to within-situation stimuli, to the process of misinterpretation. The erroneous appraisal of threat may be conceptualized as being a function of a variety of *reasoning* processes, which may be subject to errors or biases in processing. *Reasoning bias* refers to the tendency to formulate certain conclusions in a ubiquitous fashion across situations and environmental contexts (Harvey et al., 2004). A number of reasoning biases have been implicated in the etiology and maintenance of anxiety disorders. These will be discussed below.

### Emotional reasoning

The *emotional reasoning heuristic* (Schwarz and Clore, 1988) refers to the tendency for anxiety sensations to be used to infer or confirm threat. No direct evidence of emotional reasoning in flying phobia exists. A novel study by Arntz et al. (1995) demonstrated that individuals with a variety of anxiety disorders (including other forms of Specific Phobia) judged hypothetical (fear-relevant) situations as more dangerous when they were given information concerning an anxious response. Arntz and colleagues suggested that emotional reasoning may play a causal role in the persistence of anxiety disorders. As noted above, the occurrence of anxiety sensations in flying phobia (which may be cued simply by the environmental effects of air-travel; Jaffee, 2005) are believed to cue threat perception in many flying phobics. Thus, future research seeking to determine whether emotional reasoning operates within flying phobia might provide important clues regarding why the perception of threat persists in the absence of aversive outcomes.

### Anticipatory processing

Many individuals with flying phobia will experience heightened anxiety in the hours, days, and weeks preceding a flight (e.g., Wilhelm and Roth, 1997). Given that anticipatory fear and anxiety regarding flying has been established, it is surprising that little research has investigated the impact of worry on flying anxiety and behavior. We were only able to identify three studies investigating worry and flying, which suggested that self-reported worry related to flying predicted one's willingness to fly (in the aftermath of the 9/11 terrorist attacks; Bergstrom and McCaul, 2004) and also level of anxiety (Aitken et al., 1981; Martinussen et al., 2011).

The rationale for considering worry in relation to flying phobia is that worry generates anxiety and is considered to be self-perpetuating (e.g., Borkovec et al., 2004; Leigh and Hirsch, 2011). The nature of worry may also be important. For instance, verbal-based worries result in greater depletion of working memory capacity, as compared to imagery-based worry, which in turn is believed to perpetuate the worry response (Leigh and Hirsch, 2011). We were unable to identify any research which examined the form of worry typically experienced by flying phobics.

Related to worry, although conceptually distinct, is individual perception of the probability of negative outcomes occurring. Individuals with anxiety disorders typically overestimate: (i) the probability of negative (i.e., fear-relevant) events occurring relative to controls (Gilboa-Schechtman et al., 2000; Jones and Menzies, 2000); and (ii) the level of anxiety they will experience in a given situation (e.g., Salkovskis et al., 2003). These findings have not been replicated in flying phobia; however, a paucity of research has been conducted in this context. Wilhelm and Roth (1997) found that individuals with flying phobia did not demonstrate significantly different estimates regarding the likelihood of plane crashes or number or deaths as a result of commercial plane accidents compared to non-flying-phobics. However, such estimates were made in response to questions, which were not worded to describe personally-relevant outcomes (e.g., “When wings flex in turbulence they may snap off”). Möller et al. (1998) investigated the cognitive responses of 15 fearful flyers and seven non-fearful flyers through asking individuals to list their thoughts in response to six hypothetical scenarios associated with the fear of flying and six non-anxiety-provoking scenes. The fear of flying group reported higher subjective ratings of anxiety in both experimental and control scenes and reported negative cognitions conceptualized as reflecting *awfulising* (i.e., catastrophizing) and low frustration tolerance. Additionally, the phobic group's cognitions reflected more negative outcomes and an underestimation of their coping abilities relative to the control group. Such findings may be interpreted as consistent with the pattern of overestimation of likelihood of negative outcomes seen across anxiety disorders (e.g., Öst and Csatlos, 2000). Consequently, it would be helpful for future research to establish whether flying phobics rate the perceived likelihood of themselves being involved in negative flight-related outcomes more highly than non-flying phobics.

### Expectancy and covariation biases

An *expectancy bias* refers to the experimentally demonstrated cognitive bias whereby higher self-report ratings of expectancy for a threatened aversive event (e.g., an electric shock) are reported (or measured through physiological response) when presented with fear-relevant stimuli as compared to non-fear-relevant stimuli (Amin and Lovibond, 1997). An expectancy bias has been demonstrated within flying phobia as well as a number of anxiety disorder presentations (Mühlberger et al., 2006). *Covariation bias* refers to the tendency for individuals to overestimate the degree of association between a fear-relevant stimulus and a designated aversive event (Amin and Lovibond, 1997). This bias is demonstrated when fear relevant and non-fear relevant stimuli undergo an equivalent number of pairings with an aversive event (e.g., electric shock) but the fear relevant stimulus is perceived to be a stronger predictor of future aversive events. The tendency to detect an illusory correlation between a fear-relevant stimulus and aversive outcome has been suggested to be due to fear-relevant stimuli being subject to more extensive processing then non-fear relevant stimuli, thus, strengthening this association (Tomarken et al., 1989). Covariation bias has been demonstrated in flight phobics, where covariation estimates regarding the presentation of flight-related images and aversive outcome are significantly higher for flying phobics than non-flying phobics (Pauli et al., 1998). However, one study reported that flying phobics did not exhibit a covariation bias in regards to threat-related pictures (Mühlberger et al., 2006). If expectancy and covariation biases do operate within flying phobia this would suggest that individuals with a fear of flying are more likely than non-phobics to expect an aversive outcome when presented with threat-related cues (with this expectation itself increasing arousal), and will also display a greater tendency to associate flying experiences with aversive outcomes even when these variables are not necessarily correlated. Such biases would, therefore, contribute toward generating apprehension when presented with flying-related cues (or internal threat cues) and contribute to the perceived validity of subjective fears. Further investigation of these biases, whether they operate in real-world settings and whether they can be impacted upon through intervention, would be of significant value.

### Fallacious hypothesis testing and post-event processing

Aside from expectancy and covariation bias, little attention has been paid to cognitive or reasoning biases within flying phobia. Across anxiety disorders, individuals have been demonstrated to display a greater tendency toward negative interpretations of *ambiguous stimuli*, compared to controls, where stimuli may be relevant to the feared object/outcome (e.g., Amir et al., 1998; Richards et al., 2001). A fear-confirming interpretation bias has been demonstrated experimentally in disorders such as SAD (e.g., Vroling and De Jong, 2009); however, empirical support for this finding has yet to be found across each anxiety disorder. Such a bias might be hypothesized to operate in flying phobics in flying situations whereby situational stimuli (e.g., observing the plane's wings move, the expressions or behavior of other passengers or air crew), or interoceptive information, are interpreted in a manner consistent with situational danger appraisals, even where such stimuli do not connote any actual threat.

*Confirmation bias* describes the tendency to preferentially process information congruent with pre-existing beliefs regarding threat and vulnerability, which serves as confirmatory evidence of this threat (e.g., de Jong et al., 1997). Similarly, memory systems may display a propensity for recollection of information congruent with negative predictions, while ignoring non-congruent information (e.g., Mansell and Clark, 1999) contributing to erroneous pre- and post-intervention processing. Once again, this may be hypothesized to be present in flying phobia whereby news reports and videos regarding air crashes confirm a heightened evaluation of danger relating to flying or where the experience of brief turbulence confirms the lack of safety associated with flying and the perceived probability of an aversive outcome. The investigation of cognitive biases and reasoning processes within flying phobia would be of value as the identification and correction of such biases may be important steps in reversing the processes which maintain the appraisal of theat.

## Mental imagery

Over the last two decades, a growing body of research has demonstrated that mental imagery plays a significant role in the generation and maintenance of psychological distress (Brewin et al., 2010). Mental imagery refers to mental events that are experienced “as like having a sensory experience in the absence of a physical sensory stimulus” (Holmes et al., 2015, p. 1), which often contain visual imagery but may be related to any sensory modality. Whilst some authors have hypothesized a role for imagery in contributing to distress in flying phobia (e.g., Bunn, 2013) no empirical investigation, to date, has been carried out into the role of imagery in flying phobia and it is unclear if, and how, individuals with flying phobia experience mental imagery within fear-relevant situations.

A number of studies have demonstrated the occurrence of emotional mental imagery across various mental health problems (Holmes and Mathews, 2010) and there is evidence that mental images elicit greater affective response than verbal-based appraisals (Brewin et al., 2010). Indeed, there is evidence that mental imagery contributes directly to the maintenance and exacerbation of threat in SAD (e.g., Hirsch et al., 2004). Given that sources of internal threat (i.e., interoceptive cues perceived as threat) have been implicated as being of significant importance in flying phobia (Bogaerde et al., 2012), assessing the role of imagery would be an important avenue for future research to explore.

Holmes et al. (2015) suggested that more research concerning mental imagery and psychological distress is required, highlighting the potential importance of mental images of imagined events in the future. The terms *flashforwards* (Holmes et al., 2015) and *episodic future thought* (Szpunar, 2010), have been used to describe the process of envisioning potential future events, which have the potential to be experienced as intrusive. Flashforwards may be hypothesized to be a potential source of threat for flying phobics, whereby one mentally travels forward in time to an imagined flying-related catastrophe and experiences distress congruent with such imagery. Such an image may be hypothesized to confirm subjective threat as the generation of future imagery has been demonstrated to increase individuals' estimates of the probability of these imagined events occurring in reality (e.g., Carroll, 1978). In addition to confirming a flying scenario as dangerous, this may serve to ensure individuals fail to attend to information inconsistent with their appraisals of flying experiences as threatening.

Episodic future thought has been discussed as a facet of *autonoetic consciousness*, which refers to the capacity for mental time travel forwards to imagined events or backwards to remembered events (Arnold et al., 2011). This ability (which is believed to vary in degree across individuals; Arnold et al., 2011) may, therefore, be viewed as a cognitive vulnerability toward the confirmation of threat if remembered events or episodic future thoughts are consistent with feared outcomes or appraisals of danger, vulnerability to harm or uncontrollability. A priority for research into flying phobia would, therefore, be to investigate mental imagery and autonoetic consciousness in flying phobics in anticipation of flying and in response to fear-relevant stimuli.

## Memory

The role of memory in flying phobia, and anxiety disorders generally, is not sufficiently understood. Whilst intimately linked to reasoning and attentional biases, the process by which threat-relevant information is recalled may be of significant importance in maintaining anxiety responses (Harvey et al., 2004). Wilhelm and Roth (1997) suggested that memory bias may contribute to an illusory correlation between aversive experiences and flying. Only one study was identified by this review which evaluated memory in relation to the fear of flying. Bogaerde et al. (2012) reported that individuals with flying phobia demonstrated significantly greater recall for internal threat words (i.e., concerning aversive physiological outcomes) on a dichotomous listening task compared to a control sample. Notably, both groups demonstrated equivalent recall for external threat related words which suggests that, within this small sample (*N* = 25), memory bias for threatening information was demonstrated for anxiety-related threat words but not in the recall of external threat words (e.g., turbulence). These findings are consistent with the wider anxiety disorder literature (Harvey et al., 2004). Anxious individuals are believed to selectively retrieve information, which can retrospectively appear to confirm their feared outcome (Clark, 1999). Consistent with this notion, findings suggest that selective explicit memory biases for threat-relevant material may be present across anxiety disorders (e.g., Lundh and Öst, 1996; Mansell and Clark, 1999; Paunovic et al., 2002). The importance of a memory bias in threat perception may be significant. The *availability heuristic* describes a processing bias whereby the estimation of the likelihood of an event is influenced by the relative availability and/or accessibility of related memories (Tversky and Kahneman, 1973; Harvey et al., 2004) and there is evidence that the retrieval of threat-relevant outcomes may contribute to the perceived likelihood of feared outcomes occurring (e.g., Jones and Menzies, 2000). This research may suggest that spontaneous within-situation recall of distressing flying experiences (including direct aversive experiences or vicarious experiences such a news reports of plane crashes) will serve to heighten anxiety and increase the perceived probability of the feared outcome. Such recall could also involve the retrieval and ‘reliving’ of anxious states during previous plane flights, which serves to heighten anxiety and increase avoidance and/or safety-seeking behaviors (again reflecting autonoetic consciousness).

There is some evidence that individuals with anxiety disorders may avoid concern-related memories (Watts and Dalgleish, 1991; Wenzel and Holt, 2002), which may contribute to individuals continuing to predict a feared outcome even when the feared outcome has not occurred in the past. Given the potential importance of memory in the maintenance of perceived threat, future research should aim to assess retrieval biases (selective and avoidant) of flying phobics exposed to flying-related cues and evaluate whether individual characteristics (such as autonoetic experience) influence the manner in which memory impacts on phobic responses.

## Safety-seeking and counter-productive strategies

The strategies individuals perform to prevent or minimize the likelihood of feared outcomes and/or alleviate anxiety are believed to play a central role to the maintenance of anxiety disorders (Clark, 1999). It is, therefore, surprising that very little data exists to inform an understanding of what strategies are employed by flying phobics to manage their anxiety and minimize perceived threat. In anxiety disorders, these strategies can largely be categorized as *escape and avoidance* and *within-situation safety-seeking behaviors*, which are employed within anxiety-inducing situations to minimize threat (e.g., neutralizing behaviors, increasing self-focused attention, emotion-regulation strategies; Salkovskis, 1991; Harvey et al., 2004). The latter may be further categorized into *overt safety behaviors* (i.e., those which manifest as observable behaviors) and *covert safety behaviors* (i.e., those strategies which are non-observable such as thought suppression or replacement; Wells, 1997; Harvey et al., 2004).

Within-situation safety-seeking behaviors, as well as situational escape and avoidance, are considered to be counter-productive in the maintenance of anxiety disorders as they prevent the disconfirmation of perceived threat and the belief that the feared outcome will occur in the absence of such strategies (whether such fears relate to external events or the consequences of cognitive or somatic dyscontrol). These strategies are believed to account for the fact that individuals may have experiences which demonstrate that their feared outcome will not occur, yet do not experience a decrease in future fear responses to anxiety-provoking stimuli (Salkovskis, 1991). The process by which behaviors result in the failure to disconfirm perceived threat may be pertinent in the case of flying phobia, where commonly feared catastrophic outcomes (e.g., plane crashes or social humiliation) do not occur when sufferers fly and yet their anxiety response may be undiminished on a future flight. Strategies that are perceived to lead to the reduction of anxiety (e.g., the abandonment of the anxiety-inducing situation) are reinforced given that the reduction of anxiety typically leads to the misinterpretation that threat is reduced or averted only by the performance of that strategy (Salkovskis et al., 1999).

There is evidence that many individuals with flying phobia engage in avoidance of flying and report using anxiolytic medication and/or alcohol to help manage the anxiety symptoms when they do fly (Wilhelm and Roth, 1997). If these individuals have negative predictions regarding the consequences of not engaging in such behaviors, then these behaviors may be conceptualized as safety-seeking. Research demonstrates that many individuals with flying phobia avoid flying entirely (Nousi et al., 2008). Such individuals report higher levels of anxiety associated with flying and it has been suggested that this subgroup of flying phobics may tend to over-predict the magnitude and intensity of their anxiety and display general tendencies toward avoidance (Nousi et al., 2008). The only study we identified that explicitly evaluated the use of within-situation strategies in response to flying was conducted by Kraaij et al. (2003) who examined the relationship between cognitive coping strategies and anxiety. They found that flying phobics (*N* = 261) who had sought treatment for the fear of flying reported using strategies such as focussing on planning (e.g., how to manage the flight), rumination (e.g., going over thoughts and feelings in one's head) and “putting in perspective” (e.g., playing down the seriousness of events). More notably, they found that greater use of self-blame, rumination, acceptance and/or catastrophizing (i.e., explicitly emphasizing the terror of the experience) were all associated with higher levels of subjective anxiety related to flying. This finding suggests that cognitive responses within flying situations may play a significant role in exacerbating anxiety responses and that certain cognitive strategies may be considered maladaptive. The potential impact of cognitive strategies in flying phobia is highlighted in the findings of Girodo and Roehl (1978) who examined the impact of different treatment strategies on participant response to exposure to a stressful real-world flying scenario. They found that flying phobics who had received training in positive self-talk (i.e., mentally rehearsing statements reflecting positive coping relating to stressful events) reported less subjective anxiety than individuals who had not received this training. This result, again, suggests that cognitive strategies employed in flying situations may have a significant impact on anxiety.

As stated above, flying phobia has been argued to be a heterogeneous disorder with different primary fears driving the phobic response (McNally and Louro, 1992). Consequently, there would be expected to be an array of different types of behaviors exhibited which aim to minimize threat (e.g., thought suppression aimed at avoiding threatening imagery; breathing oriented-behaviors aiming to alleviate panic symptoms). However, the presence and nature of overt and covert safety behaviors in flying phobia has not been explored. Indeed, it is arguable that the only behavior explicitly targeted by the majority of flying phobia treatment programs is avoidance (e.g., Öst et al., 1997). The majority of evidence-based treatments for anxiety disorders seek to identify and work with safety-seeking behaviors as a primary component of the interventions (e.g., Clark et al., 1994, 2006). The importance of addressing within-situation safety behaviors is emphasized by the fact that focusing solely on reversing situational avoidance has been demonstrated to be inferior to treatments which aim to address all safety-seeking behaviors in disorders such as SAD (e.g., Clark et al., 2006).

The use of safety-seeking strategies to escape and/or minimize perceived threat has been demonstrated across anxiety disorders (e.g., Newth and Rachman, 2001; Stangier et al., 2006; White et al., 2006) and non-performance of safety behaviors on exposure to feared situations has been found, across anxiety diagnoses, to result in reduced belief in the probability of feared-outcomes in future exposure to threat in anxiety difficulties such as panic disorder and claustrophobia (Salkovskis et al., 1999; Sloan and Telch, 2002).

Understanding the use of covert safety-seeking behaviors may be of significant importance in flying phobia. For instance, there is evidence that the covert strategies of thought suppression (e.g., Tolin et al., 2002) and emotional suppression (Campbell-Sills et al., 2006) may prolong the experience of aversive emotions and increase the incidence of unwanted thoughts. Whilst attempts to suppress emotion and threatening cognitions (e.g., crash-related imagery) may be hypothesized to be strategies which anxious fliers would be likely to use, there has been no research carried out which demonstrates this contention. The tendency to utilize use of suppression may be conceptualized as the counter-point to trait mindfulness. Trait mindfulness is conceptualized as an adaptive dispositional attribute reflected by present-focused attention (Weinstein et al., 2009). Individuals with high levels of trait mindfulness are argued to take an accepting, non-judgmental stance toward their experience, and are able to view their thoughts and emotions as passing mental events (i.e., a decentering position), rather than accurate representations of reality (Shapiro et al., 2006). Whilst not strictly a strategy in of itself, higher trait mindfulness has been associated with lower use of counterproductive emotion regulation strategies (Tamagawa et al., 2013), and it may, therefore, be further hypothesized that trait mindfulness may be a protective variable against anxiety when flying. If trait mindfulness is a protective factor in flying anxiety then mindfulness-based techniques may be a valuable avenue for flying phobia interventions to explore.

The identification of common safety-seeking behaviors and their role in the maintenance of threat perception has formed the basis of many evidence-based treatments targeting specific anxiety disorders and, consequently, a more comprehensive understanding of their role within flying phobia is needed. Research into flying phobia could, therefore, seek to: (i) gain a comprehensive understanding of the within-situation safety-seeking strategies used by flying phobics, with particular attention given to the use of covert safety behaviors; (ii) experimentally evaluate whether manipulation of certain safety-seeking strategies impacts on anxiety and the perception of threat; and (iii) incorporate this understanding into existing treatment paradigms to more effectively target key processes of maintenance.

## Anxiety sensitivity and other pertinent constructs

*Anxiety sensitivity* (AS) is conceptualized as a cognitive vulnerability characteristic reflecting fear of anxiety and anxiety-related sensations, arising from beliefs that anxiety sensations have harmful physical, psychological, and/or social consequences (Reiss, 1991). The high levels of comorbidity between flying phobia and panic symptomology (McNally and Louro, 1992), as well as evidence of anxiety sensations as sources of threat in flying phobia (Wilhelm and Roth, 1997), has led to anxiety sensitivity receiving significant investigation in relation to the fear of flying.

Anxiety sensitivity, as measured by self-report questionnaire, is present across anxiety disorders, (Taylor et al., 1992) and may be a risk factor for anxiety problems (Feldner et al., 2006). Research from the wider anxiety disorder literature suggests that higher levels of anxiety sensitivity may be associated with increasing fears about experiencing anxiety sensations and the use of cognitive or behavioral strategies to escape internal sensations (Kashdan et al., 2008).

AS has been demonstrated to play a potentially important role in the maintenance of flying phobia. Whilst some research has found that individuals with high anxiety sensitivity do not report a stronger increase in distress in response to phobic stimuli (e.g., flying-related videos) than those individuals with low anxiety sensitivity (Busscher et al., 2010), findings from a number of studies indicate a potentially important role for anxiety sensitivity in flying phobia. For example, research utilizing self-report questionnaires within the general (i.e., subclinical) population (Bogaerde and De Raedt, 2008) and within flying phobics (Bogaerde and De Raedt, 2011) has demonstrated that AS moderates the relationship between somatic sensations and flying anxiety, where somatic symptoms significantly predict flight anxiety in individuals with higher AS scores. Busscher et al. (2013) examined the relationship between AS, flight anxiety and physiological arousal during *in vivo* exposure therapy. They did not find a significant moderating effect of AS on the relationship between self-reported somatic sensations and flight anxiety but found that changes in heart rate and parasympathetic activity during exposure displayed a stronger association with changes in reported flight anxiety for high AS participants than for those with low AS. Collectively, this research demonstrates that anxiety-related beliefs may play a significant role in contributing to the fear of flying. Furthermore, such beliefs may represent a cognitive vulnerability toward flying phobia given that flying will naturally elicit a number of somatic symptoms, which may be interpreted as threatening due to anxiety-related beliefs, leading to increased anxiety and avoidance (Bogaerde and De Raedt, 2008).

Zvolensky and Forsyth (2002) demonstrated that anxiety sensitivity is predictive of body vigilance (i.e., scanning for internal threat) in individuals without anxiety disorder diagnoses. Anxiety sensitivity predicts avoidance of emotionally salient events (Wilson and Hayward, 2006) driving behavioral avoidance and preventing the opportunity to engage in behavior that could disconfirm anxious predictions (Fedroff et al., 2000). The potential relevance of this finding for flying phobia is clear. Perhaps the most pertinent research to consider in relation to flying phobia was conducted by Kashdan et al. (2008) who demonstrated that anxiety sensitivity is associated with emotional suppression strategies and higher levels of physiological arousal and worry in a community sample. As discussed above, experimental results suggest that individuals who actively attempt to avoid or suppress anxiety experience poorer recovery from negative affect and increased physiological arousal compared to individuals who do not engage in suppression (e.g., Campbell-Sills et al., 2006). Anxiety sensitivity and the use of emotional suppression strategies may be of significant importance in the etiology and maintenance of flying phobia.

In addition, cognitive constructs associated with anxiety in other disorders would benefit from being explored in relation to flying phobia. Constructs such as *intolerance of uncertainty* (i.e., the “predisposition to react negatively to an uncertain event or situation, independent of its probability of occurrence and of its associated consequences,” Ladouceur et al., 2000, p. 934) and *thought-action fusion* (i.e., the belief that having particular thoughts may make the outcomes reflected in these thoughts more likely to manifest; Shafran et al., 1996) have been mostly associated with increasing severity of anxiety symptomology in GAD and OCD, respectively, but may be hypothesized to be relevant to the fear of flying. These beliefs have been demonstrated to be present across diagnoses (Starcevic and Berle, 2006) and, if found to be associated with flying anxiety, may be important to understand and target within treatment. Determining whether individual beliefs reflecting these constructs play any role in flying phobia would contribute to the understanding of how the fear of flying is maintained.

## Discussion

This review has evaluated a number of areas considered pertinent in the maintenance of anxiety disorders. The findings suggest that limited investigation has been conducted concerning the possible involvement of the majority of these processes in relation to flying phobia. It might be argued that research into flying phobia has been hampered somewhat by the fact that it is conceptualized as a specific phobia within the DSM-IV/DSM-5 and, as such, a relatively traditional conditioning-based understanding of the disorder has prevailed. The research described suggests that a number of cognitive processes may be involved in the maintenance of flying phobia; however, many potentially pertinent variables have yet to be investigated.

Given the current status of the literature, any attempt to outline a comprehensive, *empirically*-derived account of the processes which maintain flying phobia must be considered untenable. We will, therefore, propose a theoretical model of the processes that we hypothesize maintain flying phobia. This model is not an account of the etiology of flying phobia (for a discussion of etiology see Oakes and Bor, 2010a) but of the processes that may operate to perpetuate the fear of flying.

### Proposed model

Based on the literature reviewed and the wider anxiety disorder literature, we hypothesize that the fear of flying is driven by the experience of perceived threat related to the likelihood of negative flying-related outcomes (external threat) and/or fears related to the consequences of anxiety sensations (internal threat) experienced within a flying situation. Such perceived threat reflects a process of misinterpretation, which is maintained because there is a failure to disconfirm feared outcomes even where experiential evidence indicates that flying does not lead to catastrophic outcomes. The failure to disconfirm perceived threat occurs because:

1. *Selective attention toward threat cues* (within the internal and/or external environment) may create a hypervigilance toward threat, increase focus on internal sensations and may contribute to missing cues indicating safety.
2. *Cognitive biases* contribute toward the tendency to overestimate the likelihood of negative outcomes associated with flying and to selectively process information consistent with threatening appraisals. Additionally, attentional bias toward interoceptive information may lead to increased likelihood of making internal attributions for events and the use of internal sensations to infer danger (emotional reasoning).
3. *Pre-existing beliefs* contribute toward the tendency to interpret threat-related cues (i.e., triggers) as threatening. Such beliefs may concern: (a) the nature and consequences of triggers of threat; (b) the consequences of anxiety symptoms (i.e., anxiety sensitivity); (c) the nature and consequences of not being in control of one's situation and/or affective responses; and (d) the subjective ability to cope with the uncertain outcomes associated with flying. Such beliefs may be strengthened by the fact that:
4. *The non-occurrence of feared outcome may be attributed to the performance of cognitive or behavioral strategies* including escape, avoidance or overt or covert safety-seeking behaviors (e.g., suppression of emotions and thoughts). Beliefs regarding the performance of those behaviors (or the consequences of non-performance of those behaviors) may contribute to their continued performance and failure to disconfirm perceived threat.
5. *Future-oriented mental imagery and/or selective recall of threatening information* (autonoetic experience) may serve as confirmation of threat and exacerbate an individual's anxiety response. This may operate within flying situations and when perceived threat is experienced in the form of anticipatory negative recurrent future-oriented thought (i.e., worry).

**Figure 1:**
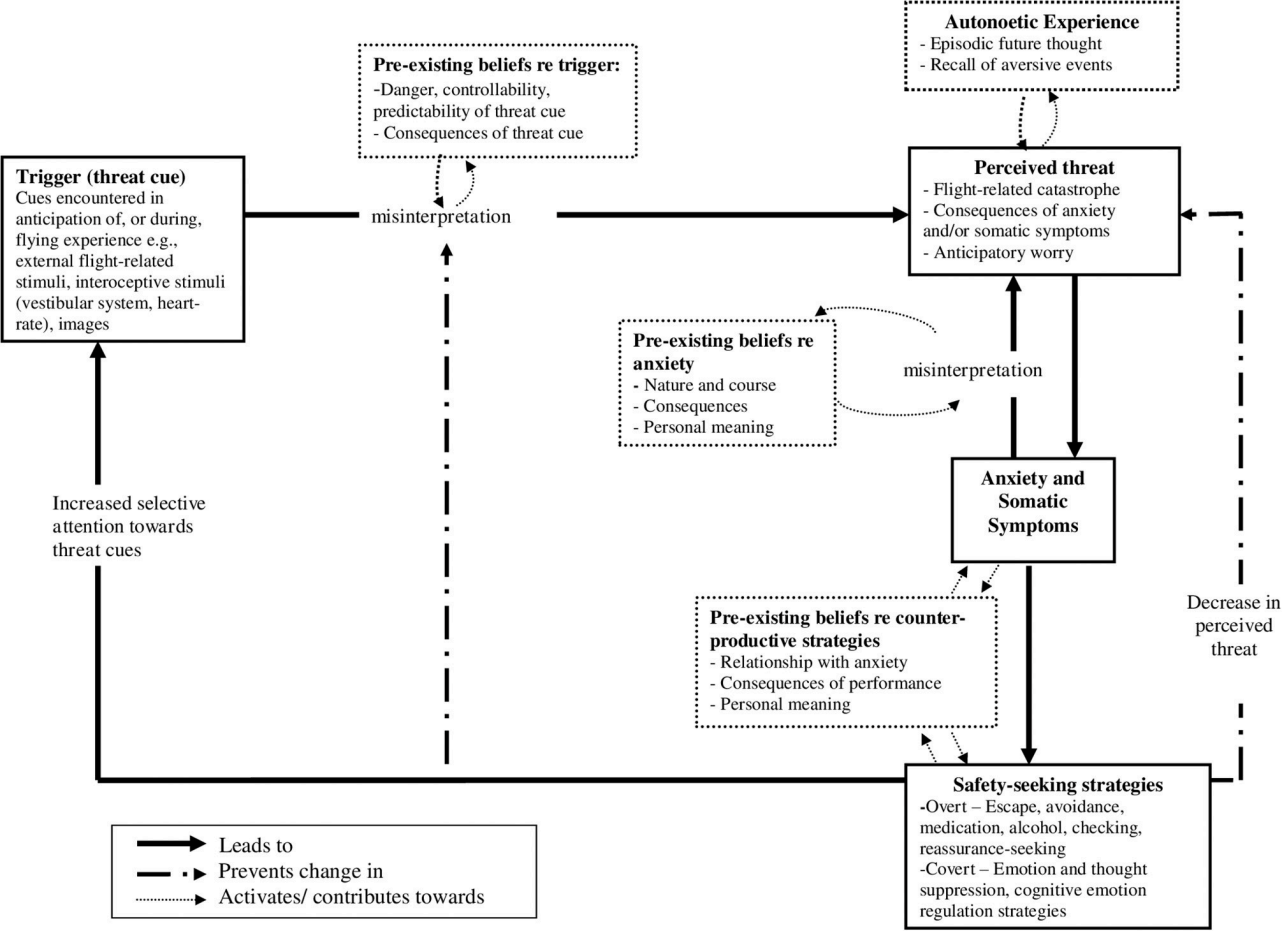
**Cognitive behavioral model of hypothesized processes which maintain flying phobia (adapted from McManus et al., 2015)**.

A diagrammatic model depicting these hypothesized maintenance processes is presented in Figure 1. The elements in each of the five areas noted above are not discrete and can be seen to overlap conceptually. Furthermore, as noted within the arrows depicted in Figure 1, each element contributes toward the maintenance and/or exacerbation of the other elements and multiple feedback loops may be hypothesized to exist. Consequently, once an individual has appraised a single flying experience as threatening, the model suggests that multiple elements may contribute to perpetuating this perceived threat and associated anxiety response. The processes proposed within the model are not novel when considered individually in relation to the wider anxiety disorder literature as the majority have been highlighted as being central processes involved in the maintenance of anxiety disorders (e.g., Clark, 1999; Harvey et al., 2004; McManus et al., 2015). However, little effort has been made to elucidate how such processes *collectively* contribute to the maintenance of anxiety in flying phobia and to propose a cognitive behavioral theoretical maintenance model. The diagrammatic model presented in Figure 1 has been adapted from the transdiagnostic anxiety model of McManus et al. (2015) and McManus and Shafran (2014), which was originally derived from an evaluation of the maintenance processes present across anxiety disorder diagnoses. Therefore, this model may serve to outline some of the key mechanisms that maintain flying phobia and which could be evaluated within future research. The lack of empirical research concerning these variables within flying phobia means that the presence or absence of these purported maintenance mechanisms, in relation to the fear of flying, has yet to be determined. More significantly, there may be some highly pertinent features of flying phobia that would be considered diagnosis-specific but which remain to be identified.

### Areas for future research

This review has highlighted a number of important areas for future research and emphasized that research concerning a number of potentially pertinent variables in the maintenance of flying phobia is non-existent or at a nascent stage. Consequently, research regarding the maintenance of flying phobia may need to adhere to first principles and adopt a grounded model of theory and treatment development (Clark, 2004). This model may include qualitative investigation targeted at the subjective experience of flying phobics, with specific reference to cognitive processes such as attention, imagery and the use of strategies to manage anxiety and minimize threat. A further area for future research to pursue would be to investigate individual cue-reactive responses to flying stimuli.

Cue-reactivity may be defined as responses to environmental stimuli (i.e., “cues”; e.g., turbulence on an aeroplane), which include fluctuations in affect, cognition and physiology (Rock and Kambouropoulos, 2007). A cue-reactivity paradigm might be used to investigate flying phobia whereby flying phobics are assessed at baseline for flight anxiety and, subsequently, exposed to a neutral cue (e.g., a video of a couple having a picnic in a park) and, finally, a flight scenario cue (e.g., the same couple traveling on an aeroplane) ^1^. A cue-reactive effect occurs if there is a statistically significant increase in cue-reactive flight anxiety from neutral cue to flight scenario cue, while controlling for baseline flight anxiety.

The cue-reactivity paradigm may be used to investigate flight phobics' *phenomenological* responses to a flight scenario cue. For example, a quantitative retrospective phenomenological assessment instrument, such as the *Phenomenology of Consciousness Inventory* (PCI, Pekala, 1991), may be used to quantify variables such as imagery, internal dialogue, rationality and volitional control. The PCI provides two types of data: (1) mean (average) intensity values for each PCI major dimension; and (2) the strength of “coupling” or association (i.e., the “pattern structure” or state of consciousness; SoC) among the various PCI major dimensions derived from a squared correlation matrix (Pekala et al., 1986). Thus, the PCI might be used to identify and compare the cue-reactive SoCs of flight phobics vs. non-flight phobics. This would allow one to identify which phenomenological sub-systems are activated by a flight scenario cue and, thus, may be related to cue-reactive flight anxiety.

Relatively little research has evaluated whether specific personality characteristics contribute toward the etiology or maintenance of flying phobia. Van Gerwen et al. (2003) suggested that individuals with personality disorders (primarily from the cluster C/anxiety domain) reported higher levels of pre-treatment fear of flying than those without such personality pathology. Further research into personality constructs and the fear of flying may, therefore, also be of benefit. Finally, it may be helpful to evaluate the contribution of dispositional traits to cue-reactive anxiety. There is evidence that individuals with flying phobia have higher levels of trait anxiety than non-phobics (Wilhelm and Roth, 1997). Additionally, it would be helpful to investigate trait variables such as trait mindfulness, autonoetic consciousness (described above) and trait absorption. Trait absorption may be defined as “a disposition for having episodes of “total” attention that fully engage one's representational (i.e., perceptual, enactive, imaginative, and ideational) resources” (Tellegen and Atkinson, 1974, p. 268). Generally speaking, trait absorption may be conceptualized as situated engrossment in a particular stimulus. It might be expected that flying phobics who are higher “absorbers” will become ensconced in threatening cues in a flying situation. Thus, trait absorption may be hypothesized to moderate the effect of flying-related fears and imagery on cue-reactive flight anxiety. However, a significant body of research is required in order to determine whether such highly tentative hypotheses are borne out.

## Conclusion

The literature reviewed indicates that flying phobia is a highly prevalent disorder that causes significant distress and impairment. The processes that maintain the disorder remain to be fully understood and a number of the key characteristics and maintaining processes identified across anxiety disorder diagnoses have yet to be investigated in relation to flying phobia. This review proposed a hypothetical model of the processes that maintain flying phobia which outlined variables which may be key to the perpetuation of the disorder. We suggest that the status of the current understanding of flying phobia is partly attributable to the fact that it has not received the breadth and scope of research afforded to other anxiety disorder presentations. We would, therefore, hope that our review serves to highlight the need for a broad range of research to be conducted which seeks to elucidate the processes that maintain and exacerbate anxiety associated with flying.

## Author contributions

GC and AR conceived the paper. GC wrote the first draft of the manuscript and both authors contributed to and have approved the final manuscript.

### Conflict of interest statement

The authors declare that the research was conducted in the absence of any commercial or financial relationships that could be construed as a potential conflict of interest.

## Footnotes

^1^We note that in addiction research this sequence of cue presentation (i.e., baseline → neutral cue → e.g., alcohol cue) has been found to produce the most reliable and robust estimates of cue-reactivity (Monti et al., 1987).
